# Hypothalamic *Crh*/*Avp*, Plasmatic Glucose and Lactate Remain Unchanged During Habituation to Forced Exercise

**DOI:** 10.3389/fphys.2020.00410

**Published:** 2020-05-15

**Authors:** Angel Toval, Francisco Vicente-Conesa, Paloma Martínez-Ortega, Yevheniy Kutsenko, Nicanor Morales-Delgado, Daniel Garrigos, Antonia Alonso, Bruno Ribeiro Do Couto, Miroljub Popović, José Luis Ferran

**Affiliations:** ^1^Department of Human Anatomy and Psychobiology, Faculty of Medicine, University of Murcia, Murcia, Spain; ^2^Institute of Biomedical Research of Murcia, Virgen de la Arrixaca University Hospital, University of Murcia, Murcia, Spain; ^3^Department of Histology and Anatomy, Faculty of Medicine, University of Miguel Hernández, Sant Joan d’Alacant, Spain; ^4^Department of Human Anatomy and Psychobiology, Faculty of Psychology, University of Murcia, Murcia, Spain

**Keywords:** running wheel, stress response, familiarization, incremental test, motor activity, paraventricular nucleus, stress biomarkers

## Abstract

It has been demonstrated that physical activity contributes to a healthier life. However, there is a knowledge gap regarding the neural mechanisms producing these effects. One of the keystones to deal with this problem is to use training programs with equal loads of physical activity. However, irregular motor and stress responses have been found in murine exercise models. Habituation to forced exercise facilitates a complete response to a training program in all rodents, reaching the same load of physical activity among animals. Here, it was evaluated if glucose and lactate – which are stress biomarkers – are increased during the habituation to exercise. Sprague-Dawley rats received an 8-days habituation protocol with progressive increments of time and speed of running. Then, experimental and control (non-habituated) rats were subjected to an incremental test. Blood samples were obtained to determine plasmatic glucose and lactate levels before, immediately after and 30 min after each session of training. *Crh* and *Avp* mRNA expression was determined by two-step qPCR. Our results revealed that glucose and lactate levels are not increased during the habituation period and tend to decrease toward the end of the protocol. Also, *Crh* and *Avp* were not chronically activated by the habituation program. Lactate and glucose, determined after the incremental test, were higher in control rats without previous contact with the wheel, compared with habituated and wheel control rats. These results suggest that the implementation of an adaptive phase prior to forced exercise programs might avoid non-specific stress responses.

## Introduction

Physically active people have a reduced risk of developing non-communicable diseases such as obesity, osteoporosis, type 2 diabetes, or heart diseases (Dishman et al., 2012; Ortega et al., 2016; Ruegsegger and Booth, 2017; Warburton and Bredin, 2017; World Health Organization [WHO], 2018). Increasing evidence has shown strong correlations between physical activity and mental health, facilitating neuroplasticity, improving brain function, and preventing mental disorders such as depression, Parkinson or Alzheimer disease (Cotman and Berchtold, 2002; Smith and Zigmond, 2003; Dishman et al., 2006; Hotting and Roder, 2013; Cooney et al., 2014). However, only a reduced number of studies in animal models were dealing with the causal mechanisms by which physical activity produces health benefits (Toval et al., 2017).

Reproducible conditions are essential in order to understand the neurobiological mechanisms of physical activity in rodent models. Animals are required to undergo similar loads of exercise, avoiding irregular motor performance and non-specific stress responses (Kregel et al., 2006; Leasure and Jones, 2008; Toval et al., 2017; Rudeck et al., 2020). Nevertheless, voluntary running is the most used exercise model in rodents; and the animals are rarely exposed to the equal intensities and volumes of running (Van Praag et al., 2005; Kregel et al., 2006; Leasure and Jones, 2008; Creer et al., 2010; Marlatt et al., 2012). These individual differences can be avoided using forced models such as treadmill or motorized wheels. However, around 10% of the rodents reject running in forced models; a situation that can be surpassed by applying an exercise habituation (HAB) protocol (Jasperse and Laughlin, 1999; Koch and Britton, 2001; Kregel et al., 2006; Toval et al., 2017). HAB to exercise is an adaptive period preceding the main training phase, highlighted by a progressive increase of speed and time of running. Toval et al. (2017) found that an 8-days HAB protocol, with an increase of speed from 5 to 9 m/min, improves the motor performance in all of the animals subjected to forced running wheels. Some authors proposed that forced exercise, like the HAB program, produces non-specific stress responses (Moraska et al., 2000; Dishman et al., 2006; Yanagita et al., 2007; Lin et al., 2012; Morgan et al., 2015). Although these hypotheses have been shown inconclusive.

Acute exercise is considered a stress condition, since it stimulates the hypothalamus-pituitary-adrenal (HPA) axis and induces metabolic and endocrine changes to maintain homeostasis under the new physiological conditions (Raastad et al., 2000; Leal-Cerro et al., 2003; Soria et al., 2015; Chen et al., 2017). The hypothalamic paraventricular nucleus (PVN) is at the beginning of the HPA axis and releases CRH and/or AVP neurochemical molecules during stress responses. CRH parvocellular neurons induces ACTH in the pituitary gland that stimulates glucocorticoid production through the adrenal gland. On the other hand, AVP magnocellular neurons are involved in circuits related with physical and psychological stress increasing the sympathetic-adrenal and catecholaminergic responses (Geerling et al., 2010; Morgan et al., 2015; Hernández et al., 2016; Godoy et al., 2018). According to Yanagita et al. (2007), the number of C-FOS/CRH positive cells in the PVN is increased during forced exercise (Timofeeva et al., 2003; Yanagita et al., 2007). These changes promote energy utilization that readies the organism for the fight-or-flight response by increasing heart rate, blood pressure or plasmatic glucose and lactate concentrations (Tan et al., 1992; Moraska et al., 2000; Chennaoui et al., 2002; Andersen et al., 2004; Kawashima et al., 2004; Soya et al., 2007; Yanagita et al., 2007; Romero Peñuela et al., 2011; Chen et al., 2017; Godoy et al., 2018). Plasmatic glucose and lactate levels have been considered useful markers of stress response after acute exercise (Raastad et al., 2000; Saito and Soya, 2004; Soya et al., 2007; Bórnez et al., 2009; Romero Peñuela et al., 2011; Garcia-Alvarez et al., 2014; Rezaei et al., 2017). Fluctuations of these physiological responses depend on the different types and intensities of exercise. For example, forced exercise models in rodents have been described to cause higher and prolonged stress responses compared with voluntary paradigms (Yanagita et al., 2007; Hayes et al., 2008; Griesbach et al., 2012).

Thus, the aim of the present study was to determine whether plasmatic glucose and lactate levels varies during an exercise HAB protocol. Additionally, plasmatic glucose and lactate changes were evaluated during an incremental exercise test; and the *Crh* and *Avp* mRNA expression were quantified after the HAB program. Our data show that plasmatic concentrations of glucose and lactate do not present any changes throughout each session of HAB, with a tendency to decrease at the end of the protocol. Finally, rats without previous contact with the wheel show significantly higher plasmatic levels of glucose and lactate after an incremental test.

## Materials and Methods

Experimental procedures were carried out following the guidelines of the animal care and use described by the Spanish (1201/2005) and the European Union (86/609/EEC) directives approved by the Ethics Commitee of the University of Murcia.

### Animals and Experimental Groups

Adolescent male Sprague-Dawley rats (Laboratory Animal Facilities at the University of Murcia) were housed in groups of three rats per cage in a room at 21–23°C and 55 ± 5% of relative humidity. Food and water were provided *ad libitum*. The light cycle was kept in a 12:12 h light/dark (dark period from 8AM to 8PM). At the age of 20 days, all the animals were randomized and assigned to either a group that received an exercise HAB protocol, a wheel control (WC) group, or a cage control (CC) group as described below:

1. HAB group (*n* = 11). Animals from this group were subjected to a HAB training protocol in a forced running wheel system. To determine plasmatic lactate and glucose, *n* = 5 animals were used. For *Crh* and *Avp* mRNA quantification, *n* = 6 animals were used.
2. CC (*n* = 11). The animals remained in the cages while the experimental group was running. To determine plasmatic lactate and glucose, *n* = 5 rats were used. For *Crh* and *Avp* mRNA quantification, *n* = 6 animals were used.
3. WC (*n* = 5). The animals remained in blocked running wheels while the experimental group was running during the HAB sessions.

### Exercise Habituation Protocol and Incremental Test

From postnatal day 26, animals of the experimental group were exposed to an 8-days exercise HAB protocol in a forced running wheel system (Lafafyette-Campdem, 80805A model, dimensions 129.54 × 45.47 × 42.93 cm) as described in Toval et al. (2017). Rats were handled and pinched in the tail daily for 5 days before the beginning of the protocol to get familiarized with the experimental procedure (Table 1).

**TABLE 1:** Schedule of the exercise habituation protocol.

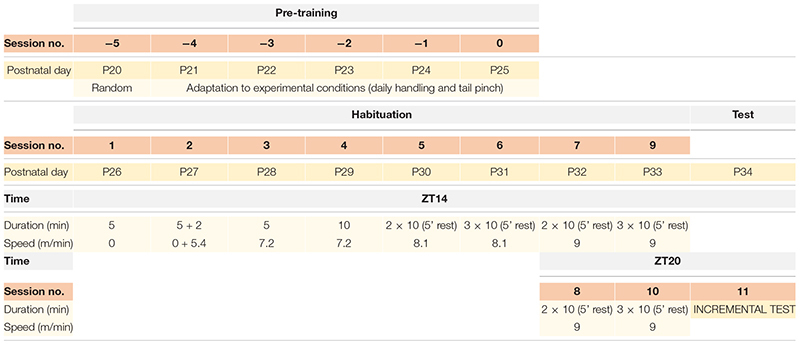

*The speed and duration of each session are described. All the sessions were performed at Zeitgeber time 14 and 20 (2 and 8 h after lights were off, in a 12-h light/12-h dark cycle).*

After 24 h of the last session of the HAB protocol, animals from the three groups were exposed to an incremental exercise test, as described in Toval et al. (2017) (Table 1). Two additional groups, HAB (*n* = 6) and CC (*n* = 6) were sacrificed just at the expected time of the incremental test to evaluate chronic activation of *Crh* and *Avp* after the HAB protocol.

### Blood Extraction

Blood samples were collected by a small incision in the distal portion of the tail (Christensen et al., 2009). Plasmatic lactate and glucose concentrations were determined 5 min prior to the running session (A), 5 min after the running session (B), and 30 min after the running session (C). Lactate was measure by a portable blood lactate analyzer (Lactate Pro LT-1710; ARKRAY Inc., Kyoto, Japan) (Tanner et al., 2010) and glucose using a portable glucometer (One Touch Ultra 2, LifeScan, Milpitas, CA, United States) (Togashi et al., 2016).

### Reverse Transcription-PCR, Cloning and *in situ* Hybridization

Reverse transcription (RT)-PCR: cDNA fragments of *Avp* and *Crh* genes to be used for *in situ* hybridization were obtained by RT. RNA was extracted from postnatal rats with Trizol (Invitrogen, Carlsband, CA, United States, Cat. 10296-028), treated with DNaseI (Invitrogen, Cat. 18068-015) and converted to cDNA with Superscript III reverse transcriptase (Invitrogen, Cat. 18080-044). The cDNA was used as a template for PCR reactions using Taq polymerase (Promega, Cat. M8305) with the following gene-specific primers for *Avp* and *Crh* mRNA: *AvpF* 5′-ACCTATGCTCGCCATGATGC-3′, *AvpR* 5′-TTGGCAGAATCCACGGACTC-3′, *CrhF* 5′-AC CTGCCAAGGGAGGAGAAGAG-3′, and *CrhR* 5′-CAAC TGGGTGACTTCCATCTGC-3′.
Cloning: The PCR products were cloned into the pGEM-T Easy Vector (Promega, Cat. A1360) and finally sequenced by ACTI research support (University of Murcia). A fragment of 495 nucleotides for *Avp* (NCBI Acc number: NM_01692; position: 17–511) and 927 nucleotides for *Crh* (NCBI Acc number: NM_031019.1; position: 141–1068) were obtained.
*In situ* hybridization: rats were sacrificed 24 h after the HAB protocol and were perfused in freshly made 4% paraformaldehyde in 0.1 M phosphate-buffered saline (PBS, pH 7.4). Brain was extracted and sliced in coronal hypothalamic sections (50 μm) with a sliding microtome (Micron HM430). All the procedures related with the *in situ* hybridization were performed as described by Ferran et al. (2015a, b) in regards to *in situ* in cryosections.

### Two-Step Quantitative Polymerase Chain Reaction

Once the rats were sacrificed brain was extracted and hypothalamus was dissected by using a brain matrix for coronal slices. The hypothalamus dissection was performed between the optic chiasma (Bregma coordinate: +0.48) and mammillary bodies (Bregma coordinate: −4.68) removing the striatum and cortex (Paxinos and Watson, 2007). Tissues were immediately frozen in dry ice and saved at −80°C for RNA extraction. All the procedures were performed in a laminar flow hood. RNA isolation was performed using NZY kits (NZY, MB1340) and analyzed to determine the RIN score for RNA integrity (Agilent Technologies, 2100 Bioanalyzer). Before RT for complementary DNA synthesis, all the RNA samples were diluted to the same concentration. RT was made using Thermofisher reagents and protocols (Cat. 18068, 18080, and 10777). After RT, the stock cDNA was diluted in eight volumes of H_2_O before use in quantitative polymerase chain reaction (qPCR). Specific primers were designed for SYBR green qPCR assays. The following primers were used at a concentration of 1.5×: *Avp*F 5′-TGCCTGCTACTTCCAGAACTGC-3′, *Avp*R 5′-AGGGGAGA CACTGTCTCAGCTC-3′, *Crh*F 5′-TGCCAAGGGAGGA GAAGAGAGC-3′, *Crh*R 5′-CAAGGCAGACAGGGCGACAG-3′, *Hprt1*F 5′-TGTTTGTGTCATCAGCGAAAGTG-3′, *Hprt1*R 5′-ATTCAACTTGCCGCTGTCTTTTA-3′, *Rplp0*F 5′-CACCAT TGAAATCCTGAGTGATGT-3′, *Rplp0*R 5′-TGACCAGCC CAAAGGAGAAG-3′, *B2m*F 5′-CGAGACCGATGTATATGCT TGC-3′, and *B2m*R 5′-GTCCAGATGATTCAGAGCTCCA-3′.

Prior to relative qPCR, absolute qPCR analyses were performed in control and experimental conditions, in order to estimate the primers efficiencies in said conditions. A 5-point, 2-fold serial dilutions were used in the assay, starting at 20 ng of sample and ending at 1.25 ng. A SYBR green master mix from Thermofisher was used (Cat. 4367659). Each well of the qPCR plate contained 5 μl of SYBR green master mix, 1 μl of 1.5× primer and 4 μl of sample, in variable in concordance with the serial dilutions described.

The relative qPCR assays were designed to contain 5 μl of SYBR green master mix, 1 μl of 1.5× primers and 10 ng of sample (a total of 10 μl per plate well). All the biological replicates were used in the assay (*n* = 6 per group), and four technical replicates per biological replicate. Negative controls of the primers were used to check for secondary structures formed by the amplicons. All the qPCR assays were performed at 60°C annealing temperature and 95°C denaturation temperature.

### Statistical Analysis

Statistical analysis was performed using SPSS v25. All data are presented as mean and standard error of the mean. The training sessions were grouped in clusters 1–3, 4–6, and 7–9, containing the means of the data acquired in the course of 3 days in each cluster.

Statistical analysis was made using SPSS v25. The normality was tested using the Shapiro–Wilk test. Values under *p* = 0.05 were considered as non-normal. For comparisons including values that did not follow a normal distribution, the U-Mann Whitney test was used. For comparisons where all values followed a normal distribution, the independent samples *T*-test was used. Levene’s test for homoscedasticity was used to determine the significance of the *T*-test.

## Results

Sprague-Dawley rats were randomly assigned to either a HAB group, a CC group, or a WC group. An 8-days HAB protocol was developed with 10 sessions where the speed and the time of running increased progressively (Table 1). Plasmatic lactate and glucose concentrations were determined in each group: 5 min prior to the running session (A), 5 min after the running session (B), and 30 min after the running session (C). Individual values were grouped into three periods: beginning (sessions 1–3), transition (sessions 4–6), and ending (sessions 7–9) of the HAB protocol.

### Lactate Values Remain Unchanged During HAB Sessions

**FIGURE 1:**
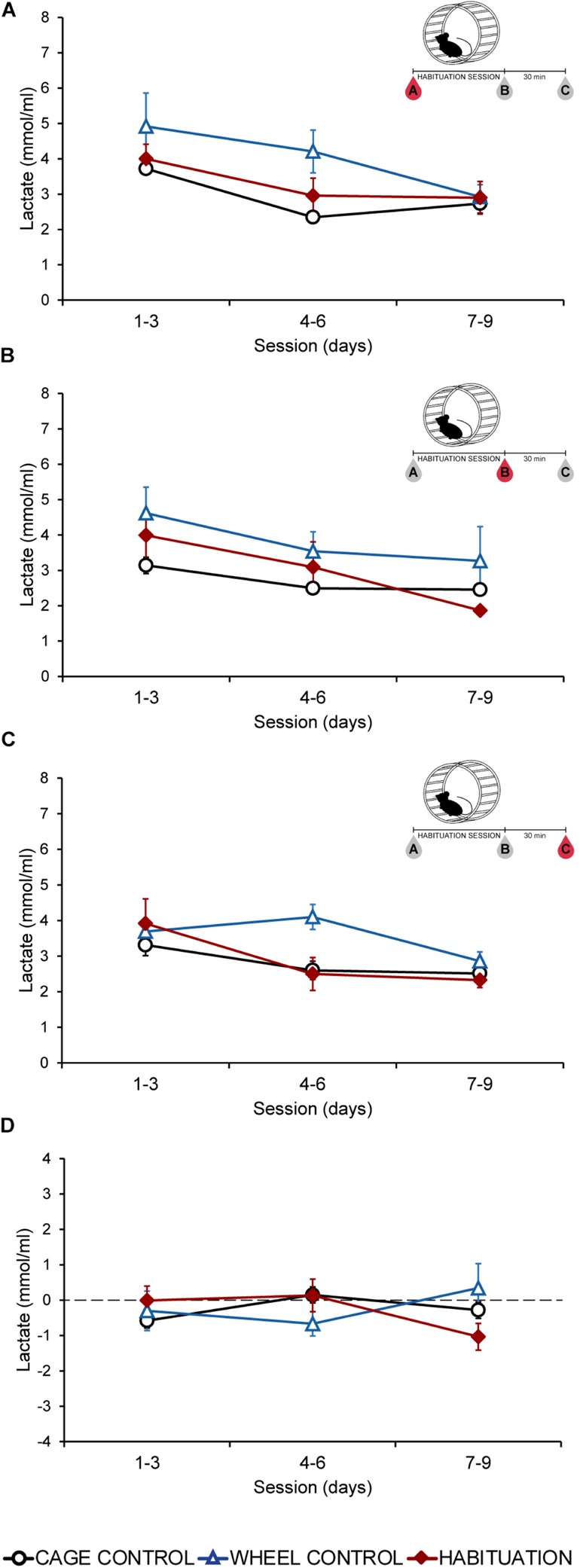
Average plasmatic lactate concentrations (mmol/ml) in SD rats throughout the habituation program comparing habituated rats, cage control, and wheel control groups. **(A)** Extraction A, 5 min before sessions. **(B)** Extraction B, 5 min after sessions. **(C)** Extraction C, 30 min after finish the session. **(D)** Difference of plasmatic lactate levels between extraction A and extraction B.

A comparison of plasmatic lactate values between habituated, WC, and CC groups in extractions A, B, or C do not show significant differences throughout most of the sessions (*p* > 0.05) (Figures 1A–C); and plasmatic lactate tend to decrease as the HAB protocol progresses; with higher values during the first 3 days (Figures 1A–C). Furthermore, to confirm that plasmatic lactate values were not increased during each session, we compared in all groups the extraction A (before running) with the extraction B (after running). Calculating the difference between A and B, no changes were found throughout all the sessions in the three groups (*p* > 0.05) (Figure 1D). HAB and control groups did not increase lactate levels.

### Plasmatic Glucose Levels Remain Unchanged During Habituation Sessions

**FIGURE 2:**
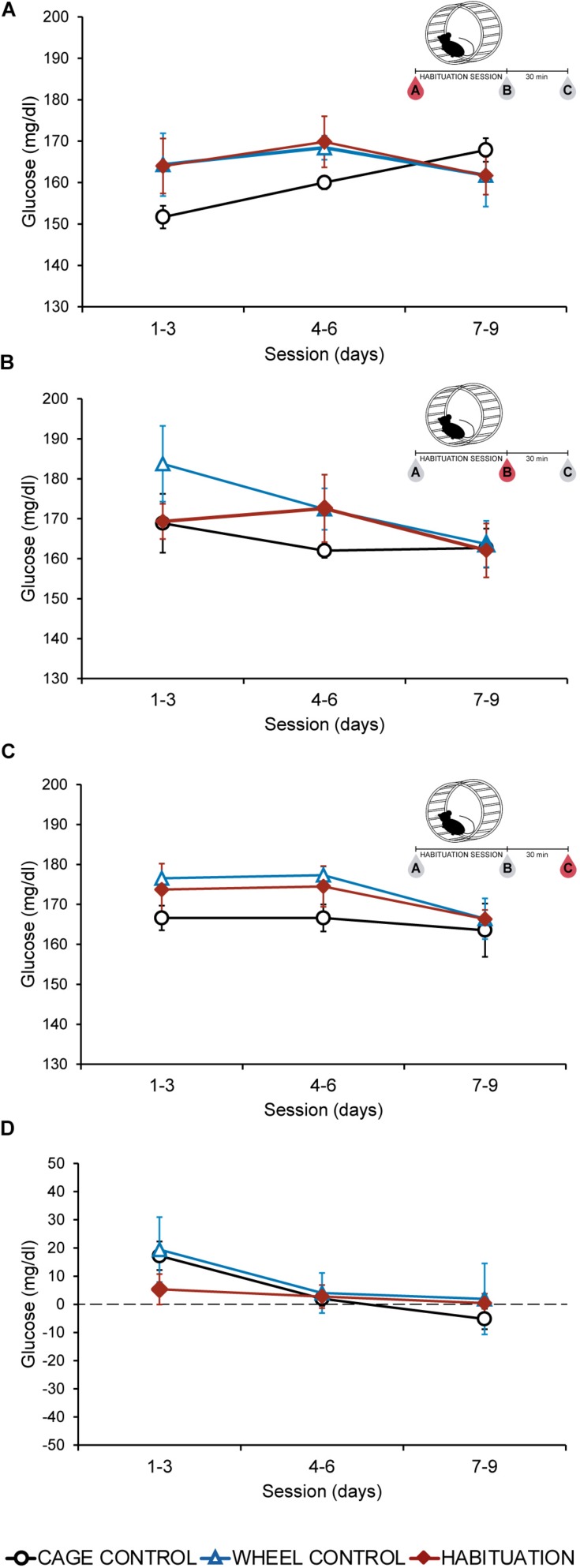
Average plasmatic glucose in SD rats throughout the habituation program comparing habituated rats, cage control, and wheel control groups. **(A)** Extraction A, 5 min before sessions. **(B)** Extraction B, 5 min after sessions. **(C)** Extraction C, 30 min after finish the session. **(D)** Difference of plasmatic glucose levels between extraction A and extraction B.

Plasmatic glucose levels did not show differences (*p* > 0.05) between habituated, WC, and CC groups in extractions A, B, or C in most of the sessions (Figures 2A–C). During the last 3 days, all the groups showed similar values (*p* > 0.05) for each extraction (Figures 2A–C). Fluctuations of plasmatic glucose obtained by subtraction of extraction A values to extraction B, remained without changes during all the protocol (Figure 2D). HAB and control groups did not increase glucose levels.

### Habituation and Wheel Contact Reduces the Lactate and Glucose Increase Produced During an Incremental Test

**FIGURE 3:**
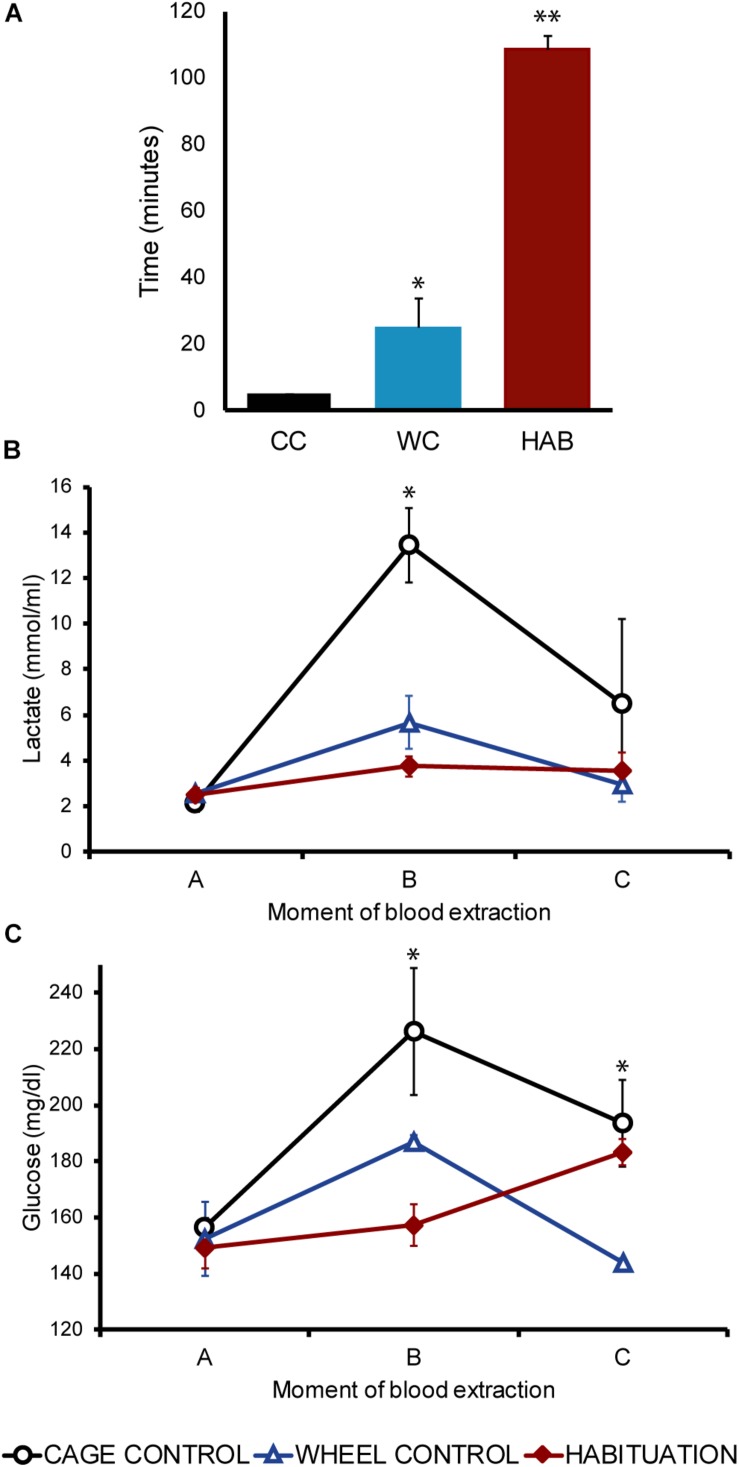
**(A)** Mean of the total time (minutes) endured during the incremental exercise test comparing cage control (CC: 5 ± 0 min), wheel control (WC: 26.5 ± 7.24 min), and habituated (HAB: 108.5 ± 4.27) rats. **(B)** Lactate concentration (mmol/ml) in SD rats before **(A)**, 5 **(B)** and 30 **(C)** minutes after the incremental test. **(C)** Glucose concentration (mmol/ml) in SD rats before **(A)**, 5 **(B)** and 30 **(C)** minutes after the incremental test.

Twenty four hours after the HAB, an incremental exercise test was carried out in all groups. The time endured by habituated rats was significantly higher compared with CC and WC groups (*p* < 0.05; HAB: 108.5 ± 4.27 min, WC: 26.6 ± 7.24 min, CC: 5 min) (Figure 3A). Blood samples were collected before (A), 5 min after (B), and 30 min after (C) the incremental test. Before starting the test (A) all groups showed similar values of plasmatic lactate and glucose (Figures 3B,C). After the test, higher values were observed in the CC group, lower values in the habituated and intermediate values in the WC (Figures 3B,C). A significant increase in lactate and glucose plasmatic values was observed in the CC group just after the test compared with habituated and WC (*p* < 0.05) (Figures 3B,C). Thirty minutes after the test, the concentration of lactate and glucose tended to decrease in all the groups, but plasmatic glucose was increased in the habituated group only (Figures 3B,C).

### Hypothalamic *Crh* and *Avp* mRNA Expression Are Not Increased After the Habituation to Forced Running Wheel

**FIGURE 4:**
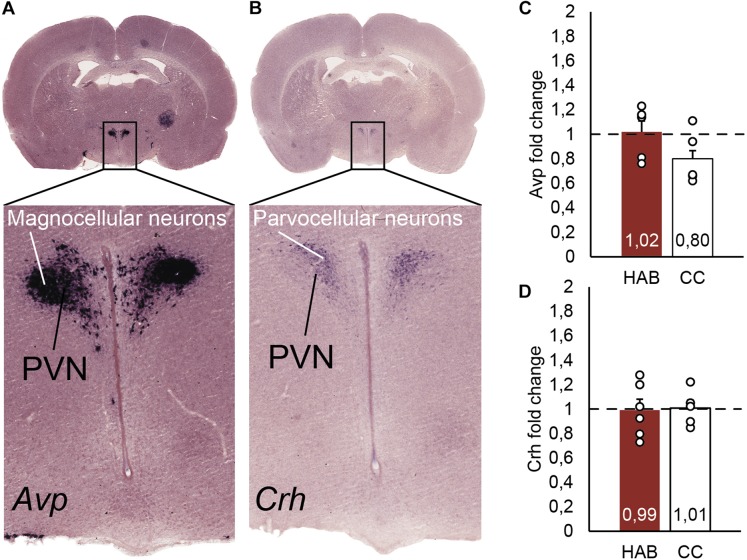
**(A)** *In situ* hybridization identifying *Avp* positive neurons in the magnocellular neurons, as part of the hypothalamic paraventricular nucleus (PVN). **(B)** *In situ* hybridization localizing *Crh* positive neurons in the parvocellular neurons, part of the hypothalamic PVN. **(C)** Real time PCR quantification of *Avp* mRNA from the parvocellular neurons in the hypothalamus. The bars represent the fold change of *Avp* comparing habituated (HAB) and cage control (CC) rats. **(D)** Real time PCR quantification of *Crh* mRNA from the parvocellular neurons in the hypothalamus. The bars represent the fold change of *Crh* comparing habituated (HAB) and cage control (CC) rats.

The PVN is characterized by parvocellular neurons expressing *Crh* mRNA and magnocellular neurons expressing *Avp* mRNA. The PVN is localized in the alar peduncular hypothalamus and is at the top of the hypothalamic-pituitary-adrenal axis, which is involved in stress responses (Sawchenko and Swanson, 1985; Morales-Delgado et al., 2011; Morales-Delgado et al., 2014; Ferran et al., 2015c). In order to determine if *Crh* and *Avp* mRNA was chronically induced after the HAB period, it was firstly confirmed by *in situ* hybridization that at postnatal 35 days *Crh* mRNA was only present in the hypothalamic region as part of parvocellular neurons of the PVN, and close to magnocellular neurons of the PVN expressing *Avp* mRNA (Figures 4A,B). Twenty four hours after finished the HAB program and at the expected time of the incremental test rats were sacrificed. The fold change of *Crh* and *Avp* mRNA was determined by two-step qPCR and no differences were found between the experimental and CC groups (Figures 4C,D). The standard curves revealed similar primer efficiencies (*Avp*, *Crh*, *Hprt1*, *Rplp0*, and *B2m*) in experimental and control conditions, and no secondary structures were detected.

## Discussion

Our results showed that plasmatic levels of the measured stress biomarkers – glucose and lactate – did not increase throughout the sessions of HAB to forced running wheels, tending to decrease toward the end of the protocol. Complementarily, it was observed that *Crh* and *Avp* mRNA expression from PVN were not increased 24 h after the HAB program. Finally, it was found that during the incremental test, higher plasmatic levels of lactate and glucose are only observed in rats without previous contact with the wheel.

Plasmatic lactate and glucose have been proven to be robust biomarkers of stress response; which are boosted after running and reach higher values when the intensity of the exercise surpasses the lactate threshold (Raastad et al., 2000; Saito and Soya, 2004; Soya et al., 2007; Bórnez et al., 2009; Romero Peñuela et al., 2011; Garcia-Alvarez et al., 2014; Rezaei et al., 2017). Once the lactate threshold is reached during running in treadmill or in forced wheel, a quick gain of glucose, lactate and ACTH can be observed (Timofeeva et al., 2003; Soya et al., 2007; Rezaei et al., 2017). The activation of the hypothalamic-pituitary-adrenal axis implies an ACTH release, which leads to a glucocorticoid effect that raises hepatic glycogenolysis, activates glycogen synthase and decreases the cellular glucose consumption. This process finally increases the plasmatic glucose levels (Stalmans and Laloux, 1979; Schneiter and Tappy, 1998; Rezaei et al., 2017). Acute stress also increases plasmatic epinephrine and norepinephrine involving the sympathetic-adrenal and catecholaminergic system, which can be related with a rise of lactate by the effects of glycogenolysis and glycolysis in the cellular cytosol (van Stegeren et al., 2010; Garcia-Alvarez et al., 2014; Godoy et al., 2018). Our results revealed that plasmatic and lactate stress biomarkers remain unchanged throughout the sessions of the HAB protocol in forced wheel, therefore discarding a stress response linked to these biomarkers during the HAB program.

Physical exercise is recognized as a stressor that activates CRH parvocellular neurons in the PVN (Timofeeva et al., 2003; Yanagita et al., 2007). Acute forced exercise in treadmill induced a higher mRNA expression of *Crh* in the PVN (Timofeeva et al., 2003). Moreover, a comparison between spontaneous and acute exercise in forced wheel running determined a higher activation of CRH neurons (C-FOS positive) in forced wheel running (Yanagita et al., 2007). In our report we aimed to determine if chronic overexpression of *Crh* and *Avp* mRNA occurred, but our qPCR results revealed that control and habituated rats have similar levels of expression. Activation of HPA axis, beginning with a CRH increase, was suggested as one of the causes responsible for the improved motor performance observed in the incremental test in habituated rats (Toval et al., 2017). Nevertheless, *Crh* mRNA levels were not over-expressed at the expected time of the incremental test, suggesting that HAB is not producing a chronic activation of HPA axis. AVP neurons projecting to the amygdaloid complex were associated with stress response modulation; and sympathetic adrenal and cathecholaminergic responses (Hernández et al., 2016; Godoy et al., 2018). According to our results this system has not been activated chronically.

Different types of training and/or intensities of exercise generate specific neuronal and physiological adaptations – a condition that justifies the need to adjust the training programs to the desired research question – (Narath et al., 2001; Dishman et al., 2006; Leasure and Jones, 2008; Kennard and Woodruff-Pak, 2012; Lin et al., 2012). However, it is frequently assumed that forced exercise models induce a non-specific emotional stress, which can be added to the physical stress arisen from high intensities of exercise. This additional source of stress may mask the proper effects derived from the physical activity (Lin et al., 2012; Morgan et al., 2015). Our data showed that rats without previous exposure to the wheels (CC) that were subjected to an incremental test, expressed higher plasmatic lactate and glucose compared with WC and habituated rats. In a similar way, previously trained rats showed a lower increase of plasmatic lactate and glucose after acute swimming compared with untrained animals (Tan et al., 1992). These results propose that, in order to avoid non-specific stress reactions in forced models, rats need to be progressively exposed to the exercise systems.

Further experiments that compare protocols using different exercise loads are needed. Furthermore, the development of experiments with implanted devices may avoid non-specific stress responses derived from the manipulations of rodents such as the methods of blood extraction. Employing devices to determine glucose or other parameters like heart rate, temperature, and motor activity will guarantee a minimal manipulation of the animals, a condition required to know the different physiological responses from a training program (Fantegrossi et al., 2013; Brown et al., 2015).

## Data Availability Statement

The data obtained are provided in the manuscript.

## Ethics Statement

All procedures with animales were reviewed and approved by the Ethics Committee of the University of Murcia.

## Author Contributions

AT and JF: study conception and design, data collection and analysis, interpretation, drafting, and revising the manuscript. FV-C and NM-D: data collection and analysis, interpretation: and revising the manuscript. YK: data collection and analysis, interpretation, drafting, and revising the manuscript. PM-O, DG, and AA: data analysis, interpretation, and revision of the manuscript. BR and MP: study conception and design, data analysis, interpretation, and revision of the manuscript. All authors have approved the final manuscript version.

## Conflict of Interest

The authors declare that the research was conducted in the absence of any commercial or financial relationships that could be construed as a potential conflict of interest.

## References

[B1] Andersen M. L. Bignotto M. Machado R. B. Tufik S. (2004). Different stress modalities result in distinct steroid hormone responses by male rats. *Braz. J. Med. Biol. Res.* 37 791–797. 10.1590/s0100-879x2004000600003 15264021

[B2] Bórnez R. Linares M. Vergara H. (2009). Haematological, hormonal and biochemical blood parameters in lamb: effect of age and blood sampling time. *Livestock Sci.* 121 200–206. 10.1016/j.livsci.2008.06.009

[B3] Brown M. B. Chingombe T. J. Zinn A. B. Reddy J. G. Novack R. A. Cooney S. A. (2015). Novel assessment of haemodynamic kinetics with acute exercise in a rat model of pulmonary arterial hypertension. *Exp. Physiol.* 100 742–754. 10.1113/ep085182 25867528 PMC13200784

[B4] Chen C. Nakagawa S. An Y. Ito K. Kitaichi Y. Kusumi I. (2017). The exercise-glucocorticoid paradox: How exercise is beneficial to cognition, mood, and the brain while increasing glucocorticoid levels. *Front. Neuroendocrinol.* 44 83–102. 10.1016/j.yfrne.2016.12.001 27956050

[B5] Chennaoui M. Gomez Merino D. Lesage J. Drogou C. Guezennec C. (2002). Effects of moderate and intensive training on the hypothalamo–pituitary–adrenal axis in rats. *Acta Physiol. Scand.* 175 113–121. 10.1046/j.1365-201X.2002.00971.x 12028131

[B6] Christensen S. D. Mikkelsen L. Fels J. Bodvarsdóttir T. Hansen A. (2009). Quality of plasma sampled by different methods for multiple blood sampling in mice. *Lab. Anim.* 43 65–71. 10.1258/la.2008.007075 19001062

[B7] Cooney G. Dwan K. Mead G. (2014). Exercise for depression. *JAMA* 311 2432–2433. 24938566 10.1001/jama.2014.4930

[B8] Cotman C. W. Berchtold N. C. (2002). Exercise: a behavioral intervention to enhance brain health and plasticity. *Trends Neurosci.* 25 295–301. 10.1016/s0166-2236(02)02143-4 12086747

[B9] Creer D. J. Romberg C. Saksida L. M. van Praag H. Bussey T. J. (2010). Running enhances spatial pattern separation in mice. *Proc. Natl. Acad. Sci. U.S.A.* 107 2367–2372. 10.1073/pnas.0911725107 20133882 PMC2836679

[B10] Dishman R. Heath G. Lee I.-M. (2012). *Physical Activity Epidemiology*, 2nd Edn, Champaign, IL: Human Kinetics.

[B11] Dishman R. K. Berthoud H. R. Booth F. W. Cotman C. W. Edgerton V. R. Fleshner M. R. (2006). Neurobiology of exercise. *Obesity* 14 345–356. 10.1038/oby.2006.46 16648603

[B12] Fantegrossi W. E. Xiao W. R. Zimmerman S. M. (2013). Novel technology for modulating locomotor activity as an operant response in the mouse: implications for neuroscience studies involving “exercise” in rodents. *J. Neurosci. Methods* 212 338–343. 10.1016/j.jneumeth.2012.10.020 23164960 PMC3629693

[B13] Ferran J. L. Ayad A. Merchán P. Morales-Delgado N. Sánchez-Arrones L. Alonso A. (2015a). Exploring brain genoarchitecture by single and double chromogenic in situ hybridization (ISH) and immunohistochemistry (IHC) in whole-mount embryos. *Situ Hybridiz. Methods* 99 61–82. 10.1007/978-1-4939-2303-8_4

[B14] Ferran J. L. Ayad A. Merchán P. Morales-Delgado N. Sánchez-Arrones L. Alonso A. (2015b). “Exploring brain genoarchitecture by single and double chromogenic in situ hybridization (ISH) and immunohistochemistry (IHC) on cryostat, paraffin, or floating sections,” in *Situ Hybridization Methods*, eds Hauptmann G. (New York, NY: Humana Press), 83–107. 10.1007/978-1-4939-2303-8_5

[B15] Ferran J. L. Puelles L. Rubenstein J. L. (2015c). Molecular codes defining rostrocaudal domains in the embryonic mouse hypothalamus. *Front. Neuroanat.* 9:46 10.3389/fnana.2015.00046 PMC4400913 25941476

[B16] Garcia-Alvarez M. Marik P. Bellomo R. (2014). Stress hyperlactataemia: present understanding and controversy. *Lancet Diabetes Endocrinol.* 2 339–347. 10.1016/S2213-8587(13)70154-2 24703052

[B17] Geerling J. C. Shin J. W. Chimenti P. C. Loewy A. D. (2010). Paraventricular hypothalamic nucleus: axonal projections to the brainstem. *J. Comp. Neurol.* 518 1460–1499. 10.1002/cne.22283 20187136 PMC2868510

[B18] Godoy L. D. Rossignoli M. T. Pereira P. D. Garcia-Cairasco N. Umeoka E. H. D. L. (2018). A comprehensive overview on stress neurobiology: basic concepts and clinical implications. *Front. Behav. Neurosci.* 12:127. 10.3389/fnbeh.2018.00127 30034327 PMC6043787

[B19] Griesbach G. S. Tio D. L. Vincelli J. McArthur D. L. Taylor A. N. (2012). Differential effects of voluntary and forced exercise on stress responses after traumatic brain injury. *J. Neurotrauma* 29 1426–1433. 10.1089/neu.2011.2229 22233388 PMC3335105

[B20] Hayes K. Sprague S. Guo M. Davis W. Friedman A. Kumar A. (2008). Forced, not voluntary, exercise effectively induces neuroprotection in stroke. *Acta Neuropathol.* 115 289–296. 10.1007/s00401-008-0340-z 18210137 PMC2668645

[B21] Hernández V. S. Hernández O. R. Perez de la Mora M. Gómora M. J. Fuxe K. Eiden L. E. (2016). Hypothalamic vasopressinergic projections innervate central amygdala GABAergic neurons: implications for anxiety and stress coping. *Front. Neural Circ.* 10:92. 10.3389/fncir.2016.00092 27932956 PMC5122712

[B22] Hotting K. Roder B. (2013). Beneficial effects of physical exercise on neuroplasticity and cognition. *Neurosci. Biobehav. Rev.* 37 2243–2257. 10.1016/j.neubiorev.2013.04.005 23623982

[B23] Jasperse J. L. Laughlin M. H. (1999). Vasomotor responses of soleus feed arteries from sedentary and exercise-trained rats. *J. Appl. Physiol.* 86 441–449. 10.1152/jappl.1999.86.2.441 9931174

[B24] Kawashima H. Saito T. Yoshizato H. Fujikawa T. Sato Y. McEwen B. S. (2004). Endurance treadmill training in rats alters CRH activity in the hypothalamic paraventricular nucleus at rest and during acute running according to its period. *Life Sci.* 76 763–774. 10.1016/j.lfs.2004.09.014 15581908

[B25] Kennard J. A. Woodruff-Pak D. S. (2012). A comparison of low- and high-impact forced exercise: effects of training paradigm on learning and memory. *Physiol. Behav.* 106 423–427. 10.1016/j.physbeh.2012.02.023 22402029 PMC3349001

[B26] Koch L. G. Britton S. L. (2001). Artificial selection for intrinsic aerobic endurance running capacity in rats. *Physiol Genomics* 5 45–52. 10.1152/physiolgenomics.2001.5.1.45 11161005

[B27] Kregel K. C. Allen D. L. Booth F. W. Fleshner M. R. Henriksen E. J. Musch T. (2006). Resource book for the design of animal exercise protocols. *Am. J. Vet. Res.* 68 583–583. 10.2460/ajvr.68.6.583

[B28] Leal-Cerro A. Gippini A. Amaya M. J. Lage M. Mato J. A. Dieguez C. (2003). Mechanisms underlying the neuroendocrine response to physical exercise. *J. Endocrinol. Invest.* 26 879–885. 10.1007/bf03345239 14964441

[B29] Leasure J. Jones M. (2008). Forced and voluntary exercise differentially affect brain and behavior. *Neuroscience* 156 456–465. 10.1016/j.neuroscience.2008.07.041 18721864

[B30] Lin T.-W. Chen S.-J. Huang T.-Y. Chang C.-Y. Chuang J.-I. Wu F.-S. (2012). Different types of exercise induce differential effects on neuronal adaptations and memory performance. *Neurobiol. Learn. Mem.* 97 140–147. 10.1016/j.nlm.2011.10.006 22085720

[B31] Marlatt M. W. Potter M. C. Lucassen P. J. van Praag H. (2012). Running throughout middle-age improves memory function, hippocampal neurogenesis, and BDNF levels in female C57BL/6J mice. *Dev. Neurobiol.* 72 943–952. 10.1002/dneu.22009 22252978 PMC3485396

[B32] Morales-Delgado N. Castro-Robles B. Ferrán J. L. Martinez-de-la-Torre M. Puelles L. Díaz C. (2014). Regionalized differentiation of CRH, TRH, and GHRH peptidergic neurons in the mouse hypothalamus. *Brain Struct. Funct.* 219 1083–1111. 10.1007/s00429-013-0554-2 24337236 PMC4013449

[B33] Morales-Delgado N. Merchan P. Bardet S. M. Ferran J. L. Puelles L. Díaz C. (2011). Topography of somatostatin gene expression relative to molecular progenitor domains during ontogeny of the mouse hypothalamus. *Front. Neuroanat.* 5:10 10.3389/fnana.2011.00010 PMC3057523 21441981

[B34] Moraska A. Deak T. Spencer R. L. Roth D. Fleshner M. (2000). Treadmill running produces both positive and negative physiological adaptations in Sprague-Dawley rats. *Am. J. Physiol. Regul. Integr. Comp. Physiol.* 279 R1321–R1329. 10.1152/ajpregu.2000.279.4.R1321 11004000

[B35] Morgan J. A. Corrigan F. Baune B. T. (2015). Effects of physical exercise on central nervous system functions: a review of brain region specific adaptations. *J. Mol. Psychiatry* 3:3. 10.1186/s40303-015-0010-8 26064521 PMC4461979

[B36] Narath E. Skalicky M. Viidik A. (2001). Voluntary and forced exercise influence the survival and body composition of ageing male rats differently. *Exp. Gerontol.* 36 1699–1711. 10.1016/s0531-5565(01)00145-0 11672990

[B37] Ortega F. B. Lavie C. J. Blair S. N. (2016). Obesity and cardiovascular disease. *Circ. Res.* 118 1752–1770. 10.1161/CIRCRESAHA.115.306883 27230640

[B38] Paxinos G. Watson C. (2007). *The Rat Brain In Stereotaxic Coordinates*, 6th Edn, Amsterdam: Elsevier.

[B39] Raastad T. Bjøro T. Hallen J. (2000). Hormonal responses to high-and moderate-intensity strength exercise. *Eur. J. Appl. Physiol.* 82 121–128. 10.1007/s004210050661 10879453

[B40] Rezaei S. Agha-alinejad H. Shamsi M. M. Jafari M. Voltarelli F. A. Naderi A. (2017). Evaluation of efforts in untrained Wistar rats following exercise on forced running wheel at maximal lactate steady state. *J. Exerc. Nutr. Biochem.* 21:26. 10.20463/jenb.2017.0040 28712262 PMC5508056

[B41] Romero Peñuela M. H. Uribe-Velásquez L. F. Sánchez Valencia J. A. (2011). Biomarcadores de estrés como indicadores de bienestar animal en ganado de carne: stress biomarkers as indicators of animal welfare in cattle beef farming. *Biosalud* 10 71–87.

[B42] Rudeck J. Vogl S. Banneke S. Schönfelder G. Lewejohann L. (2020). Repeatability analysis improves the reliability of behavioral data. *PloS One* 15:e0230900. 10.1371/journal.pone.0230900 32240211 PMC7117744

[B43] Ruegsegger G. N. Booth F. W. (2017). Health benefits of exercise. *Cold Spring Harb. Perspect. Med.* 8:a029694. 10.1101/cshperspect.a029694 PMC6027933 28507196

[B44] Saito T. Soya H. (2004). Delineation of responsive AVP-containing neurons to running stress in the hypothalamus. *Am. J. Physiol. Regul. Integr. Comp. Physiol.* 286 R484–R490. 10.1152/ajpregu.00453.2003 14630623

[B45] Sawchenko P. Swanson L. (1985). Localization, colocalization, and plasticity of corticotropin-releasing factor immunoreactivity in rat brain. *Fed. Proc.* 44 221–227. 2981743

[B46] Schneiter P. Tappy L. (1998). Kinetics of dexamethasone-induced alterations of glucose metabolism in healthy humans. *Am. J. Physiol. Endocrinol. Metab.* 275 E806–E813. 10.1152/ajpendo.1998.275.5.E806 9815000

[B47] Smith A. D. Zigmond M. J. (2003). Can the brain be protected through exercise? Lessons from an animal model of parkinsonism. *Exp. Neurol.* 184 31–39. 10.1016/j.expneurol.2003.08.017 14637076

[B48] Soria M. González-Haro C. Ansón M. López-Colón J. L. Escanero J. F. (2015). Plasma levels of trace elements and exercise induced stress hormones in well-trained athletes. *J. Trace Elem. Med. Biol.* 31 113–119. 10.1016/j.jtemb.2015.04.004 26004901

[B49] Soya H. Mukai A. Deocaris C. C. Ohiwa N. Chang H. Nishijima T. (2007). Threshold-like pattern of neuronal activation in the hypothalamus during treadmill running: establishment of a minimum running stress (MRS) rat model. *Neurosci. Res.* 58 341–348. 10.1016/j.neures.2007.04.004 17524508

[B50] Stalmans W. Laloux M. (1979). Glucocorticoids and hepatic glycogen metabolism. *Monogr. Endocrinol.* 12 517–533. 10.1007/978-3-642-81265-1_27 114752

[B51] Tan N. Morimoto K. Sugiura T. Morimoto A. Murakami N. (1992). Effects of running training on the blood glucose and lactate in rats during rest and swimming. *Physiol. Behav.* 51 927–931. 10.1016/0031-9384(92)90072-a 1514957

[B52] Tanner R. K. Fuller K. L. Ross M. L. (2010). Evaluation of three portable blood lactate analysers: lactate pro, lactate scout and lactate plus. *Eur. J. Appl. Physiol.* 109 551–559. 10.1007/s00421-010-1379-9 20145946

[B53] Timofeeva E. Huang Q. Richard D. (2003). Effects of treadmill running on brain activation and the corticotropin-releasing hormone system. *Neuroendocrinology* 77 388–405. 10.1159/000071311 12845225

[B54] Togashi Y. Shirakawa J. Okuyama T. Yamazaki S. Kyohara M. Miyazawa A. (2016). Evaluation of the appropriateness of using glucometers for measuring the blood glucose levels in mice. *Sci. Rep.* 6:25465. 10.1038/srep25465 27151424 PMC4858715

[B55] Toval A. Baños R. De la Cruz E. Morales-Delgado N. Pallares J. G. Ayad A. (2017). Habituation training improves locomotor performance in a forced running wheel system in rats. *Front. Behav. Neurosci.* 11:42. 10.3389/fnbeh.2017.00042 28337132 PMC5340750

[B56] Van Praag H. Shubert T. Zhao C. Gage F. H. (2005). Exercise enhances learning and hippocampal neurogenesis in aged mice. *J. Neurosci.* 25 8680–8685. 10.1523/JNEUROSCI.1731-05.2005 16177036 PMC1360197

[B57] van Stegeren A. H. Roozendaal B. Kindt M. Wolf O. T. Joëls M. (2010). Interacting noradrenergic and corticosteroid systems shift human brain activation patterns during encoding. *Neurobiol. Learn. Mem.* 93 56–65. 10.1016/j.nlm.2009.08.004 19695335

[B58] Warburton D. E. Bredin S. S. (2017). Health benefits of physical activity: a systematic review of current systematic reviews. *Curr. Opin. Cardiol.* 32 541–556. 10.1097/HCO.0000000000000437 28708630

[B59] World Health Organization [WHO] (2018). *Global Action Plan On Physical Activity 2018–2030: More Active People For A Healthier World.* Geneva: WHO.

[B60] Yanagita S. Amemiya S. Suzuki S. Kita I. (2007). Effects of spontaneous and forced running on activation of hypothalamic corticotropin-releasing hormone neurons in rats. *Life Sci.* 80 356–363. 10.1016/j.lfs.2006.09.027 17067638

